# Exposure to a Northern Contaminant Mixture (NCM) Alters Hepatic Energy and Lipid Metabolism Exacerbating Hepatic Steatosis in Obese JCR Rats

**DOI:** 10.1371/journal.pone.0106832

**Published:** 2014-09-15

**Authors:** Ryan J. Mailloux, Maria Florian, Qixuan Chen, Jin Yan, Ivan Petrov, Melanie C. Coughlan, Mahemuti Laziyan, Don Caldwell, Michelle Lalande, Dominique Patry, Claude Gagnon, Kurtis Sarafin, Jocelyn Truong, Hing Man Chan, Nimal Ratnayake, Nanqin Li, William G. Willmore, Xiaolei Jin

**Affiliations:** 1 Toxicology Research Division, Bureau of Chemical Safety, Food Directorate, Health Canada, Ottawa, Ontario, Canada; 2 Scientific Support Division, Bureau of Chemical Safety, Food Directorate, Health Canada, Ottawa, Ontario, Canada; 3 Nutrition Research Division, Bureau of Nutritional Sciences, Food Directorate, Health Canada, Ottawa, Ontario, Canada; 4 Hazard Identification Division, Environmental Health Science and Research Bureau, Healthy Environments and Consumer Safety Branch, Health Canada, Ottawa, Ontario, Canada; 5 Departments of Biology and Chemistry, Carleton University, Ottawa, Ontario, Canada; 6 Departments of Biology, University of Ottawa, Ottawa, Ontario, Canada; INRA, France

## Abstract

Non-alcoholic fatty liver disease (NAFLD), defined by the American Liver Society as the buildup of extra fat in liver cells that is not caused by alcohol, is the most common liver disease in North America. Obesity and type 2 diabetes are viewed as the major causes of NAFLD. Environmental contaminants have also been implicated in the development of NAFLD. Northern populations are exposed to a myriad of persistent organic pollutants including polychlorinated biphenyls, organochlorine pesticides, flame retardants, and toxic metals, while also affected by higher rates of obesity and alcohol abuse compared to the rest of Canada. In this study, we examined the impact of a mixture of 22 contaminants detected in Inuit blood on the development and progression of NAFLD in obese JCR rats with or without co-exposure to10% ethanol. Hepatosteatosis was found in obese rat liver, which was worsened by exposure to 10% ethanol. NCM treatment increased the number of macrovesicular lipid droplets, total lipid contents, portion of mono- and polyunsaturated fatty acids in the liver. This was complemented by an increase in hepatic total cholesterol and cholesterol ester levels which was associated with changes in the expression of genes and proteins involved in lipid metabolism and transport. In addition, NCM treatment increased cytochrome P450 2E1 protein expression and decreased ubiquinone pool, and mitochondrial ATP synthase subunit ATP5A and Complex IV activity. Despite the changes in mitochondrial physiology, hepatic ATP levels were maintained high in NCM-treated versus control rats. This was due to a decrease in ATP utilization and an increase in creatine kinase activity. Collectively, our results suggest that NCM treatment decreases hepatic cholesterol export, possibly also increases cholesterol uptake from circulation, and promotes lipid accumulation and alters ATP homeostasis which exacerbates the existing hepatic steatosis in genetically obese JCR rats with or without co-exposure to ethanol.

## Introduction

**Figure 1:**
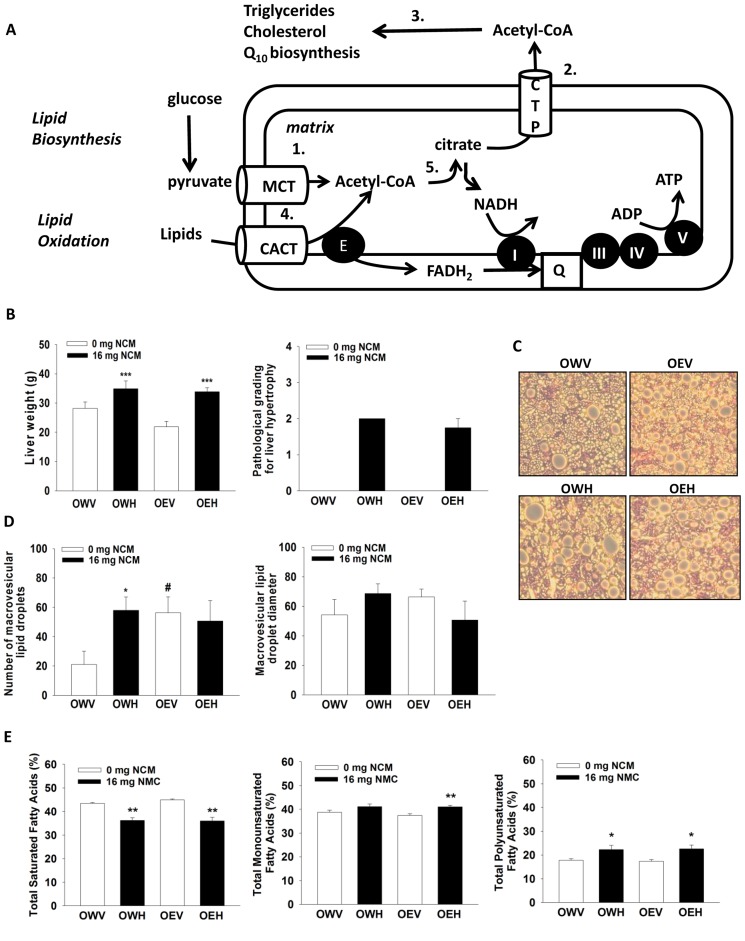
Impact of NCM treatment on liver physiology and fatty acid profile in obese JCR rats treated with or without ethanol. A) Involvement of mitochondria in hepatic lipid biosynthesis and degradation. *1*. pyruvate enters into mitochondria and is converted to acetyl-CoA in the matrix. *2*. Following the synthesis of citric acid it is exported to the cytosol from the matrix and cleaved to reproduce acetyl-CoA. *3*. Acetyl-CoA is carboxylated and then utilized for the synthesis of fatty acids which then esterify glycerol to produce triglycerides. *4*. Lipids enter the matrix and are oxidized to acetyl-CoA. *5*. Acetyl-CoA enters the TCA cycle and produces NADH which then drives oxidative phosphorylation. E: electron transport flavoprotein-ubiquinone oxidoreductase, CTP: citrate transport protein, MCT: monocarboxylate transporter, CACT: carnitine:acyl-carntine transporter. B) Liver weights, *n* = 8, means ±SEM, and pathological grading of liver hypertrophy. C) Oil red staining of lipid droplets in liver section. D) Total number of lipid vacuoles and quantification of lipid vacuole diameter. Lipid droplets number and diameter were determined using Axion Vision software. *n* = 6, means ±SEM. E) Hepatic saturated fatty acid, monounsaturated fatty acid, and polyunsaturated fatty acid levels. Fatty acids were extracted and measured by GC-MS. *n* = 8, means ±SEM. Two-way ANOVA with Tukey's post-hoc test. * denotes statistical comparison between vehicle control (V) and high dose (H) and ^#^ denotes statistical comparison between water (W) and ethanol (E) treated groups. * or ^#^, **, and *** indicate significant difference at p<0.05, 0.02, and 0.001, respectively. OWV; obese water vehicle, OWH; obese water high dose, OEV; obese ethanol vehicle, OEH; obese ethanol high dose.

The liver plays a critical role in lipid homeostasis where various metabolic enzymes and proteins work in tandem to balance fat levels in the body (**Figure 1A**). Lipid homeostasis is maintained by *de novo* lipid biosynthesis (lipogenesis and cholesterol biosynthesis) and mitochondrial β-oxidation [1]. Lipid degradation occurs in mitochondria and ultimately results in the production of ATP [2]. *De novo* lipogenesis, on the other hand, involves the commitment of acetyl-CoA produced from citric acid to the genesis of short, medium, and long-chain fatty acids which are then esterified to produce triglycerides [3]. Acetyl-CoA can also be reacted with acetoacetate to generate 3-hydroxy-3-methylglutaryl-CoA (HMG-CoA) which is used for the production of cholesterol, ubiquinone (Coenzyme Q_10_ or Q), and other steroid containing molecules [4]. Following production, lipids are then emulsified by ApoB-100 at microsomes, forming very low density lipoprotein (VLDL) which is then released by exocytosis into circulation [5]. Lipid biosynthesis and degradation do not operate simultaneously since it would lead to the futile cycling of acetyl-CoA and the loss of energy. Instead both pathways are activated and deactivated, respectively, in response to the changing energy demands and nutrient status of the body.

Maintenance of lipid homeostasis requires functional mitochondria. During β-oxidation, electrons are systematically stripped from lipids yielding NADH and FADH_2_ which then transfer electrons to Q through Complex I and electron transfer flavoprotein quinone oxidoreductase (ETF-QO) [6]. Electrons from Q are then passed through Complex III to molecular oxygen O_2_ at Complex IV. The transfer of electrons to molecular oxygen yields a transmembrane proton gradient which is used to drive ATP synthesis via Complex V [7]. Lipid biosynthesis relies on the synthesis and export of citric acid, the first intermediate of the Krebs cycle, from mitochondria. In the cytosol, citric acid is cleaved by ATP-citrate lyase to yield acetyl-CoA which is then committed to triglyceride synthesis. Disruption of mitochondrial function is often associated with hepatic accumulation of lipids and the occurrence of non-alcoholic fatty liver disease (NAFLD) and obesity [8]. NAFLD is associated with mitochondrial DNA (mtDNA) damage, oxidative stress, decreased respiratory complex protein levels and activities, and decreased mitochondrial ATP production [9], [10]. This consequently diminishes β-oxidation resulting in intrahepatic lipid accumulation and the development of micro and macrosteatosis [11]. Overconsumption of foods rich in carbohydrates amplifies hepatic lipid synthesis which can lead to development of obesity and NAFLD [12]. Chronic ethanol consumption can also lead to the accumulation of hepatic triglycerides and the onset of alcoholic fatty liver disease by inhibiting β oxidation and enhancing lipogenesis [13]. Thus, mitochondrial dysfunction can encourage diversion of substrates destined for oxidation in mitochondria towards lipid synthesis and accumulation and the onset of NAFLD.

Disruption of fatty acids and cholesterol uptake, transport, synthesis, and excretion in liver and peripheral tissues leads to development of NAFLD. Increasing evidence suggests that exposure to elevated levels of various industrial chemicals, including persistent organic pollutants (POPs) and toxic metals, can contribute to the development of NAFLD and eventually the development of toxicant-induced steatohepatitis (TASH) and liver failure [14], [15]. Chronic exposure to polychlorinated biphenyls (PCBs), lead (Pb), and mercury (Hg) are associated with NAFLD in American adults [16]. Cadmium (Cd) exposure has been associated with NAFLD in adult men and, to a lesser extent, adult women [14]. Prolonged exposure to agrochemicals and pesticides like organophosphates has also been shown to contribute to NAFLD and liver disease [17]. The capacity of these chemicals to induce NAFLD stems from their ability to disrupt mitochondrial function. *In vitro* and *in vivo* studies have conclusively shown that toxic metals and certain POPs preferentially accumulate in mitochondria, leading to oxidative damage and disruption of mitochondrial respiration [3], [18], [19]. The result is the accumulation of hepatic lipids due to a loss of mitochondrial function and capacity to engage in β-oxidation. Furthermore, hepatic mitochondria may be more prone to the toxic effects of POPs and metals since the liver is the primary site for the accumulation of these contaminants in the body.

The rates of obesity and NAFLD in the Northern populations have increased significantly in the last few decades, with contributing factors including consumption of calorie rich retail foods, departure from traditional lifestyles, and alcohol abuse. Northern populations can also be exposed to high concentrations of various environmental contaminants (**Table 1**) which may contribute to the development of NAFLD. An Inuit Health Survey conducted in 2004 and 2005 revealed that Inuit blood samples contain higher concentrations of various environmental contaminants, compared to Southern populations, including a range of PCBs, toxic metals, and organochlorines (**Table 1**) [20]. Exposure levels of Arctic populations vary tremendously however; a small percentage of Arctic populations accumulate high levels of these contaminants. The impact of an individual contaminant on NAFLD and hepatic energy homeostasis may be altered by the presence of other toxins. It also remains uncertain if the development of obesity and/or alcohol abuse has an effect on contaminant toxicity and the development of NAFLD. Here, we examined the effects of exposure to a Northern Contaminant Mixture (NCM), which was prepared based on the types and levels of contaminants found in Inuit plasma samples [20] (**Table 1**), on liver physiology, lipid, lipoprotein and cholesterol homeostasis and mitochondrial function in obese JCR rats treated with or without 10% ethanol. We observed that obese JCR rats dosed with NCM at a level relevant to some highly exposed Arctic populations developed a more severe form of macrovesicular steatosis with hepatic hypertrophy which was accompanied by decreased circulating levels of cholesterol and triglyceride and increased hepatic total cholesterol and cholesterol ester levels. The effects of NCM were amplified by co-exposure to non-toxic levels of ethanol. These effects were associated with increased hepatic expression of cytochrome P450 2E1 (CYP2E1) protein level and loss of a functional mitochondrial respiratory chain and depletion of the Q pool. Despite the loss of functional mitochondria, hepatic ATP levels were elevated in rats exposed to NCM which was due to increased creatine kinase activity and decreased utilization of ATP. The implications of our findings and how they relate to toxicant-induced hepatic steatosis (TASH) are discussed herein.

**Table 1:** Chemical composition and concentrations of the NCM as compared with those detected in Inuit plasma [20].

Contaminants	Mean levels detected in Inuit plasma samples (ng/L)	Dose (µg/kg BW)^a^
Heavy Metals		
Cadmium	3035	1461.00
Mercury (methylmercury)	10997	2410.00
Lead	39368	4972.80
Polychlorinated biphenyls (PCBs)		
99	170	47.00
138	534	190.00
146	180	61.00
153	1333	400.00
163	221	62.00
170	216	61.82
180	813	227.27
187	287	91.00
194	182	51.00
201	167	47.00
203	105	25.00
Organochlorines		
Oxychlordane	431	160.00
p,p'-DDE	3232	500.00
Trans-nonachlor	725	220.00
Pentachlorophenol	914	180.00
Toxaphene		
Parlar # 50	142	59.13
Brominated flame retardants		
Polybrominated diphenyl ethers (PBDE) IUPAC #47	72	24.00
2,3,4,6-tetrabromophenol	36	13.95
Perfluorinated compounds		
Perfluorooctanesulfonic acid (PFOS)	29000	4700.00
*Total concentrations*	*101929*	15963.97

a Based on our previous experience, to achieve certain desired serum concentrations of chemicals in animals hundreds times higher exposure levels have to be used. This is due to the removal, detoxification and excretion of chemicals by liver and kidney, and accumulation of chemicals in various organs.

## Results

### NCM exacerbates hepatic steatosis in obese rats

Obese rats were treated with a contaminant mixture found to occur in the blood of Inuits, with vehicle solvent serving as control. Rats were simultaneously treated with either water or ethanol to ascertain if alcohol had an interactive effect with the NCM. Thus, the impact of NCM treatment, in conjunction with ethanol exposure, on liver physiology and metabolism was compared between four groups; obese:water:vehicle (OWV), obese:water:high dose (OWH), obese:ethanol:vehicle (OEV), and obese:ethanol:high dose (OEH). Trace amounts of various contaminants including dichlorodiphenyldichloroethylene (DDE), pentabromodiphenylether (BDE47), and various PCBs were detected in liver (**Table 2**) and serum (**Table 3**) of the vehicle control groups. The serum levels of individual organic contaminants were in the range of geometric mean and maximum levels found in the Inuit blood [20], and were higher in the OWH than the OEH group (**Table 3**). Inversely, higher hepatic concentrations of mercury were found in the OEV and OEH than the OWV and OWH groups. Mercury levels were comparable to those found in the liver of marine mammals in Canadian arctic [21]. Hepatic levels of contaminants were multiple folds higher in NCM treated groups compared to controls. Livers of obese JCR rats exposed to NCM (OWH and OEH) weighed more than livers exposed to vehicle solvent (OWV and OEV respectively) (**Figure 1B**). Pathological analysis revealed hepatic hypertrophy in NCM-treated animals with or without alcohol treatment (**Figure 1B**). Histological analysis of liver sections revealed that OWV animals did present with NAFLD as indicated by the presence of both micro and macrovesicular lipid droplets (**Figure 1C and 1D**). NCM treatment significantly increased the number of macrovesicular lipid droplets. Chronic ethanol exposure also significantly increased the number of macrovesicular lipid droplets. The combination of NCM and ethanol did not increase the number of lipid droplets further (**Figure 1D**). No changes in macrovesicular lipid droplet diameter were recorded (**Figure 1D**). We also measured the impact of NCM on hepatic fatty acid levels. Regardless of ethanol, NCM significantly increased hepatic total monounsaturated fatty acids (MUFA), especially in the presence of ethanol, and polyunsaturated fatty acids (PUFA), but decreased total saturated fatty acids (SFA) (**Figure 1E**). NCM treatment increased hepatic total lipid contents with or without ethanol exposure (**Table 2**). These results indicate that NCM augments hepatic accumulation of lipids, which worsens steatosis in our animal model.

**Table 2:** Lipid contents and contaminant levels (ng/g wt) in the liver of obese JCR rats dosed with vehicle or NCM and treated with or without ethanol.

Contaminants	OWV	OWH	OEV	OEH
Lipid (% of wt)	33.12±2.12	37.27±1.73^a^	25.33±2.25	31.46±1.51^a^
Mercury	63.25±15.27	8727±1098.35^a^	530.50±70.36^b^	11940.48±3421.75^ ab^
DDE	2.21±0.58	3778.52±449.09 a	2.34±0.32	3193.95±422.85 a
Trans-nonachlor	0.09±0.09	29.14±3.15 a	0.17±0.11	36.82±10.75 a
BDE47	0.34±0.16	248.75±31.32 a	0.11±0.22	214.81±39.89 a
PCB 99	0.04±0.04	1232.24±167.85 a	0.00±0.00	1025.43±175.01 a
PCB 138	0.52±0.26	3265.22±370.47 a	0.34±0.17	2811.03±476.89 a
PCB 145	0.11±0.08	1322.65±145.80 a	2.21±0.50	1199.53±227.46 a
PCB 153	1.84±0.63	8062.49±996.93 a	2.22±0.50	6913.04±1340.53 a
PCB 163	0.26±0.09	1054.49±134.08 a	0.26±0.06	917.83±173.74 a
PCB 170	0.39±0.15	1783.46±206.88 a	0.59±0.13	1528.32±309.27 a
PCB 180	1.78±0.43	6418.95±680.23 a	2.15±0.40	5548.04±1045.25 a
PCB 187	0.61±0.16	2241.05±263.80 a	0.71±0.16	1943.93±390.95 a
PCB 194	0.52±0.16	3336.89±420.49 a	0.59±0.14	2751.45±543.72 a
PCB 201	0.75±0.34	2962.90±418.90 a	0.58±0.14	2285.67±490.47 a
PCB 203	0.25±0.08	1349.29±147.81 a	0.34±0.078	1140.84±253.07 a

*n* = 7–8, means ± SEM, 2-way ANOVA with Tukey's post-hoc test.

a denotes statistically significant difference between vehicle (V) control and high dose (H) groups at p<0.001.

b denotes statistically significant difference between OWV and OEV, and between OWH and OEH groups at p<0.001.

**Table 3:** Contaminant levels (ng/g wt) in the sera of obese JCR rats treated with vehicle (V) or high dose (H) NCM with (E) or without (W) co-exposure to ethanol.

Contaminants	OWV	OWH	OEV	OEH
PCP	0.035±0.024	44.747±4.722^a^	0.187±0.135	33.900±5.861^a^
TBP	0.070±0.061	0.145±0.034^a^	0.013±0.008	0.126±0.027^a^
Oxychlordane	0.009±0.009	8.900±1.692^a^	0.527±0.236	5.101±0.577^a^
trans-Nonachlor	0.000±0.000	0.927±0.116^a^	0.267±0.135	1.043±0.122^a^
PCB99	0.165±0.103	4.259±0.511^a^	0.549±0.365	4.152±1.537^a^
p,p-DDE	0.080±0.069	29.600±8.020^a^	0.546±0.493	16.171±2.285^a^
PCB146	0.000±0.000	7.993±0.549^a^	0.296±0.000	5.346±0.720^a^
PCB153	1.771±0.850	58.532±9.578^a^	1.612±0.578	32.923±4.765^a^
PCB163	0.116±0.045	7.295±0.566^a^	0.306±0.238	4.690±0.599^a^
PCB138	0.351±0.190	28.357±6.231^a^	0.649±0.332	14.470±1.953^a^
PCB187	0.000±0.000	18.038±2.762^a^	0.285±0.283	10.622±1.448^a^
BDE47	0.174±0.085	1.859±0.239^a^	0.419±0.293	1.372±0.223^a^
PCB180	1.272±0.673	43.953±2.950^a^	1.259±0.484	27.370±3.726^a^
PCB201	0.000±0.000	16.137±2.039^a^	1.031±0.000	8.713±1.187^a^
PCB170	0.000±0.000	11.929±1.135^a^	0.143±0.130	7.211±0.992^a^
PCB203	0.000±0.000	8.727±1.179^a^	0.265±0.239	4.946±0.624^a^
PCB194	0.547±0.306	16.020±2.043^a^	0.649±0.215	8.884±1.230^a^

n = 7–8, means ±SEM, 2-way ANOVA with Tukey's post-hoc test.

a denotes statistically significant difference between vehicle (V) control and high dose (H) groups at p<0.05.

### NCM decreases hepatic cholesterol and Q biosynthesis

**Figure 2:**
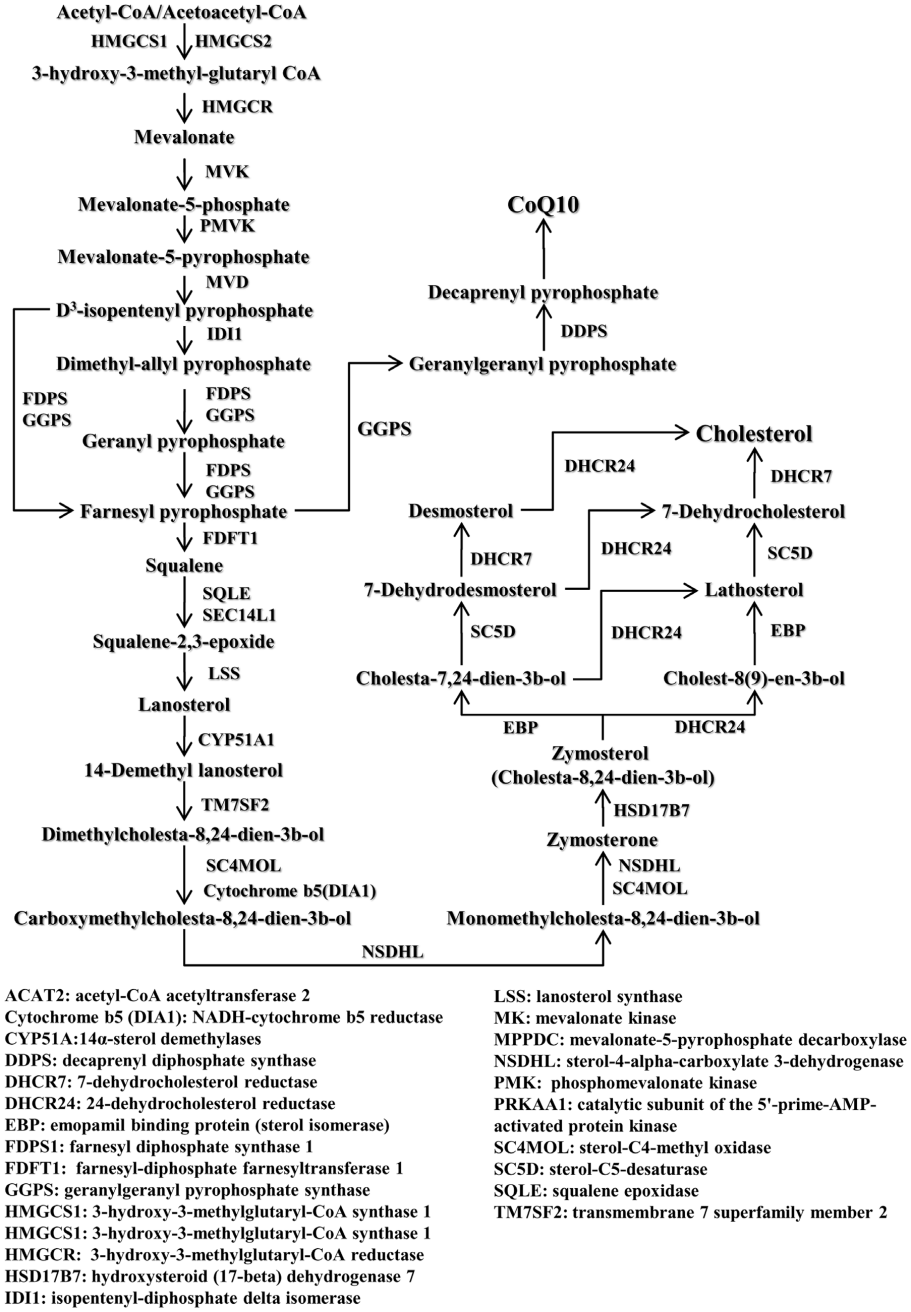
Pathways of hepatic cholesterol and Coenzyme Q_10_ (CoQ10) biosynthesis in liver.

**Figure 3:**
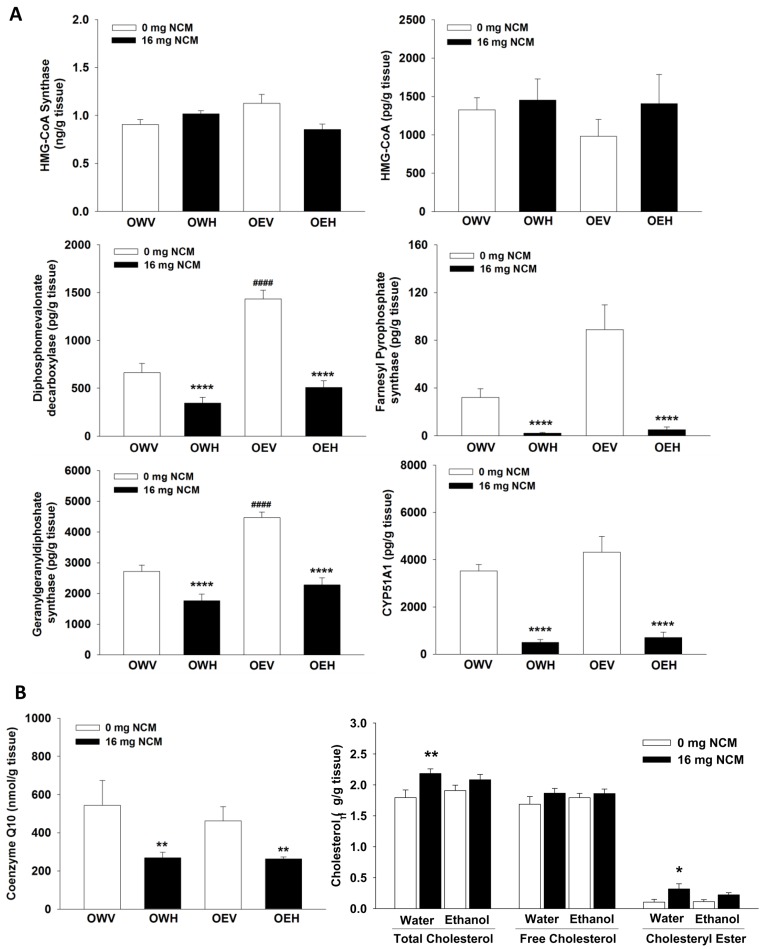
NCM treatment decreases hepatic CoQ_10_, but increases total cholesterol and cholesterol ester levels, which is associated with significant decreases in the expressions of some enzymes in the synthetic pathway of cholesterol and CoQ10. **A**) Measurement of protein levels of various enzymes involved in cholesterol and CoQ10 biosynthesis in liver homogenate. Protein levels and amount of 3-hydroxy-3-methylglutaryl-CoA (HMG-CoA) were ascertained using ELISA as described in *Materials and Methods*. *n* = 6, means ±SEM. **B**) Assessment of hepatic CoQ_10_ and free and total cholesterol and cholesteryl ester levels. Protein levels of geranylgeranyldiphosphate synthase were measured by ELISA. Absolute amounts of CoQ_10_ were measured by HPLC as described in *Materials and Methods*. Cholesterol levels were quantified using a Cholesterol/Cholesteryl Ester Detection Kit from Abcam according to instruction. *n* = 6, means ±SEM. Two-way ANOVA with Tukey's post-hoc test. * denotes statistical comparison between vehicle control (V) and high dose (H) and ^#^ denotes statistical comparison between water (W) and ethanol (E) treated groups. *, **, and **** or ^####^ indicate significant difference at p<0.05, 0.01 and 0.0001, respectively. OWV; obese water vehicle, OWH; obese water high dose, OEV; obese ethanol vehicle, OEH; obese ethanol high dose.

The synthesis of cholesterol is reliant on a number of different enzymes which produce isoprene units which are subsequently extended and cyclized, producing cholesterol (**Figure 2**). The enzyme levels of HMG-CoA synthase, which commits acetyl-CoA to steroidogenesis, were unaffected by NCM treatment and/or ethanol exposure (**Figure 3A**). We also observed no differences in the absolute levels of HMG-CoA (**Figure 3A**). Exposure to ethanol alone, with no NCM treatment (OEV), induced a significant increase in diphosphomevalonate decarboxylase (**Figure 3A**) and a trend of increase was observed for farnesyl pyrophosphate synthase. Ethanol exposure had no effect on the other enzymes that were measured. Surprisingly, significant decreases in diphosphomevalonate decarboxylase, farnesyl pyrophosphate synthase, and CYP51A1 protein levels were observed in liver extracts from obese JCR rats treated with NCM (OWH and OEH) regardless of ethanol exposure (**Figure 3A**). Thus, it can be concluded that exposure to NCM for 4 weeks leads to a profound decrease in cholesterol biosynthesis enzymes with or without co-exposed to ethanol.

These surprising observations prompted us to test the impact of NCM on hepatic Q and cholesterol levels. The biosynthesis of Q is reliant on isoprene units from the cholesterol pathway where farnesyl pyrophosphate is used to produce decaprenyl pyrophosphate, the isoprene required for the synthesis of Q (**Figure 2**) [22]. The enzyme that commits farnesyl pyrophosphate to synthesis of decaprenyl pyrophosphate, geranylgeranyl pyrophosphate synthase, was significantly decreased in obese JCR rats exposed to NCM, with or without ethanol (OWH and OEH) (**Figure 3A**). Exposure to ethanol alone (OEV) induced a profound increase in the enzyme levels of geranylgeranyl diphosphate synthase (**Figure 3A**). We next measured the absolute levels of hepatic Q. Exposure of obese JCR rats to NCM with or without ethanol (OWH and OEH) induced a significant decrease in absolute Q levels. Thus, NCM compromises *de novo* biosynthesis of Q (**Figure 3B**). Surprisingly, NCM treatment caused no change in hepatic free cholesterol levels with or without ethanol, but increased cholesterol ester and total cholesterol levels, especially in the absence of ethanol (**Figure 3B**).

### Impact of NCM on the respiratory chain

**Figure 4:**
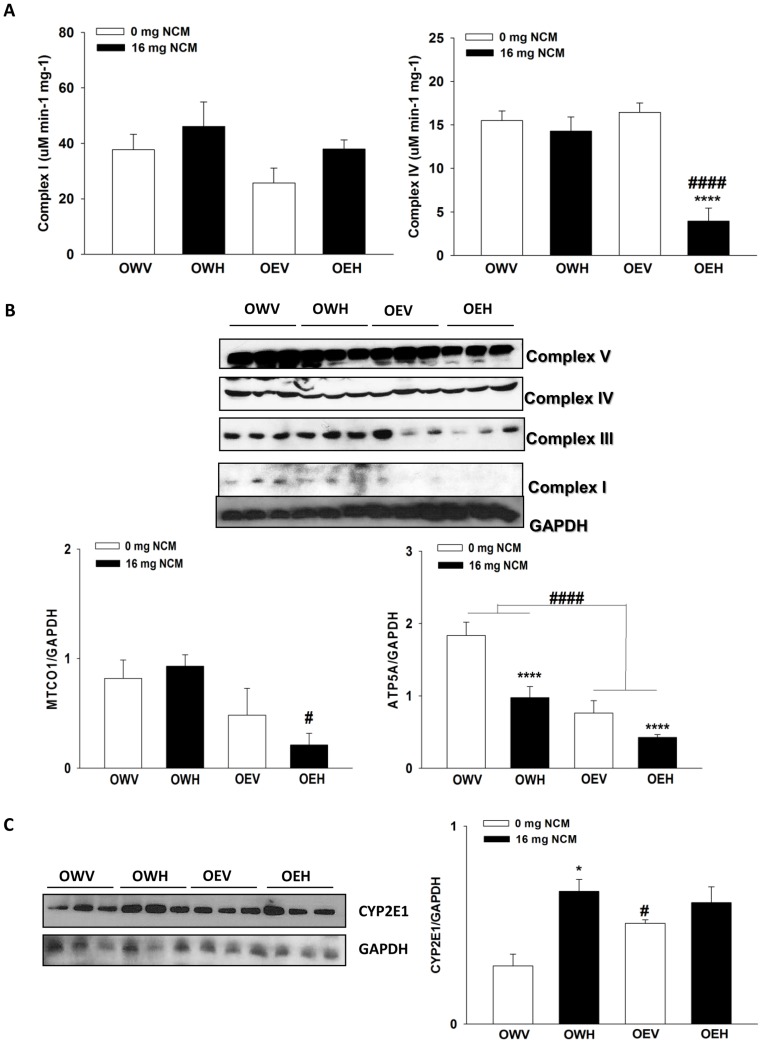
NCM treatment diminishes the activity and expression Complexes IV and V of the respiratory chain, which is associated with increased hepatic CYP2E1 protein levels. **A**) Measurement of the specific activities of Complex I (NADH:ubiquinone oxidoreductase) and Complex IV (cytochrome C oxidase) in liver homogenate. For Complex I, activities were ascertained by measuring the consumption of NADH. Complex IV activities were determined by measuring the consumption of Cyt C. *n* = 5, means ±SEM. **B**) Immunoblot analysis of respiratory complex protein content. Different respiratory complex subunits were detected simultaneously using OXPHOS MitoProfile antibodies. Membranes were then stripped and probed for GAPDH. Blots were quantified using ImageJ software and values were normalized to GAPDH loading control levels. *n* = 3, means ±SEM. **C**) Immunoblot analysis of CYP2E1 protein levels. Membranes were stripped and probed for GAPDH. Blots were quantified using ImageJ software and values were normalized to GAPDH loading control levels. *n* = 3, means ±SEM. Two-way ANOVA with Tukey's post-hoc test. *, or ^#^ and **** or ^####^ denotes P<0.05, P<0.0001 respectively. * denotes statistical comparison between vehicle control (V) and high dose (H) and ^#^ denotes statistical comparison between water (W) and ethanol (E) treated groups. OWV; obese water vehicle, OWH; obese water high dose, OEV; obese ethanol vehicle, OEH; obese ethanol high dose.

The decrease in hepatic Q levels prompted us to measure the activities of Complex I and IV. No changes in the specific activity of Complex I was observed in obese JCR rats treated with NCM, with or without ethanol (**Figure 4A**). The specific activity of Complex IV was also unaffected in obese JCR rats treated with NCM or ethanol alone (**Figure 4A**). However, Complex IV specific activity was decreased significantly in liver extracts from obese JCR rats treated with both NCM and ethanol (OEH). We next examined the protein levels of the five respiratory Complexes by immunoblotting for specific subunits of each Complex simultaneously. It is worthy to note that we were unable to detect the iron-sulfur subunit of succinate dehydrogenase (SDHB) of Complex II even when up to 45 µg of protein was loaded. All the antibodies in the MitoProfile cocktail were raised in mouse, which may explain the lack of reactivity of the antibody mixture towards JCR rat SDHB. As shown in **Figure 4B**, NDUFB8, UQCRC2, MTCO1, and ATP5A subunits of Complexes I, III, IV, and V respectively displayed profound changes in expression following exposure to NCM and/or ethanol. NDUFB8 displayed faint bands however; expression tended to decrease with NCM treatment (**Figure 4B**). In extracts from ethanol or NCM plus ethanol treated obese JCR rats (OEV and OEH respectively), the expression of NDUFB8 was completely abolished. UQCRC2 was also unaffected by NCM; however, exposure to ethanol substantially diminished the protein levels of this Complex III subunit (**Figure 4B**). Protein bands for MTCO1 seemed unaffected by NCM and/or ethanol treatment. However, band quantification and subsequent normalization to GAPDH revealed that MTCO1 was actually significantly decreased only in liver extracts from NCM plus ethanol treated obese JCR rats (**Figure 4B**). Finally, expression of ATP5A was significantly affected by NCM treatment in the absence or presence of ethanol. NCM or ethanol alone decreased ATP5A levels by ∼2-fold, which was decreased even further (∼2.5-fold) when NCM and ethanol were administered simultaneously. We also examined the impact of treatments on CYP2E1 protein levels and found it was significantly increased in NCM groups (OWH and OEH) and in rats treated with ethanol alone (OEV) (**Figure 4C**). It is important to note that the combination of ethanol and NCM did not increase CYP2E1 expression further, i.e. the combined effects of the two treatments were not additive. Thus, it can be concluded that NCM and/or ethanol exposure has a profound impact on respiratory enzyme complement and activity in liver tissue.

### NCM alters hepatic ATP homeostasis

**Figure 5:**
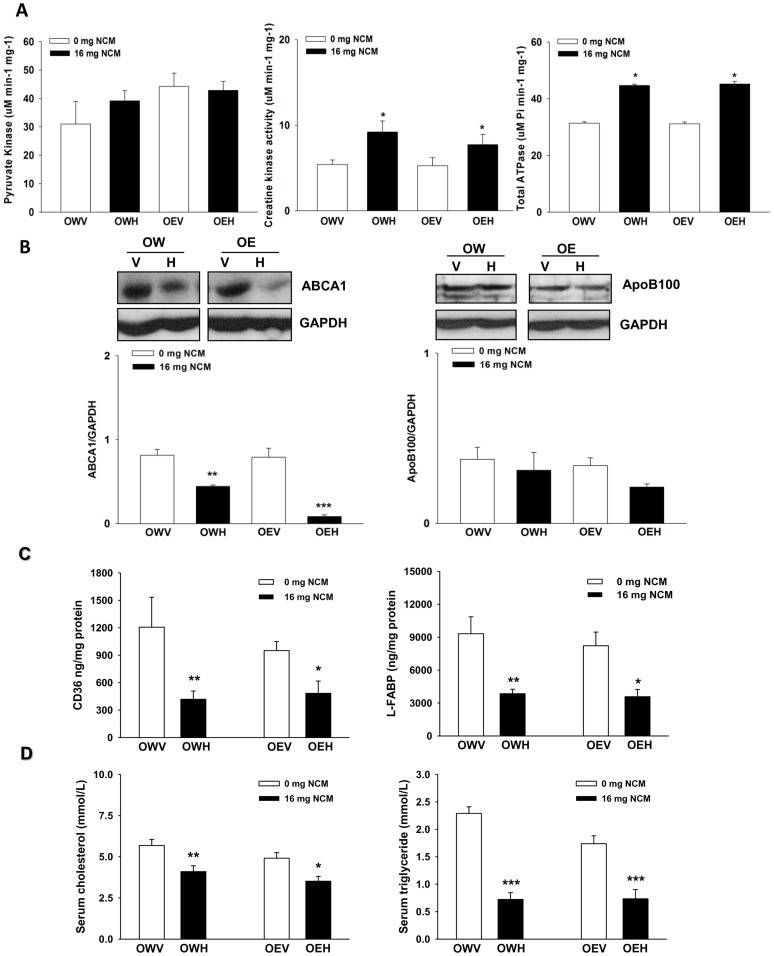
NCM exposure increases hepatic creatine kinase activity and total ATPase activity, which is associated with decreases in hepatic ABCA1, CD36, and L-FABP protein expression and circulating levels of cholesterol and triglycerides. **A**). Measurement of the specific activities of pyruvate kinase, creatine kinase, and total MDR ATPases in liver homogenate. For pyruvate kinase, activities were ascertained by measuring the consumption of NADH. Creatine kinase activities were measured by ADP production. *n* = 5, means ±SEM. Total MDR ATPases activities were measured using BD Gentest ATPase Assay kit. **B**) Immunoblot analysis of ABCA1 and ApoB-100 protein levels. Membranes were stripped and probed for GAPDH. Blots were quantified using ImageJ software and values were normalized to GAPDH loading control levels. *n* = 3, means ±SEM. **C**) Hepatic CD36 and L-FABP levels were measured using ELISA kits from MyBioSource. *n* = 6, means±SEM. **D**) Serum cholesterol and triglycerides levels were measured using the Hitachi Model 917 Multichannel Analyzer. *n* = 5, means ±SEM. Two-way ANOVA with Tukey's post-hoc test. *, **, and *** denotes P<0.05, 0.01 and 0.001 respectively. * denotes statistical comparison between vehicle control (V) and high dose (H) and ^#^ denotes statistical comparison between water (W) and ethanol (E) treated groups. OWV; obese water vehicle, OWH; obese water high dose, OEV; obese ethanol vehicle, OEH; obese ethanol high dose.

The changes in Q_10_ and mitochondrial respiratory complexes prompted us to examine the impact of NCM and/or ethanol exposure on hepatic adenosine nucleotide levels. Using HPLC, we were able to quantify the impact of NCM and/or ethanol on ATP, ADP, and AMP levels. As shown in **Table 4**, NCM treatment (OWH) induced a significant accumulation of ATP, compared to OWV, which was mirrored by significant decreases in ADP and AMP. Similarly, ATP levels in livers from obese JCR rats exposed to NCM and ethanol (OEH) were also significantly higher when compared to OEV (**Table 4**). However, in contrast to OWH, OEH did not display any changes in ADP or AMP relative to OEV. These observations, coupled with the decrease in ATP5A expression and the shifts in the expression of various respiratory enzyme complexes, prompted us to examine the activities of pyruvate kinase and creatine kinase. Both pyruvate kinase and creatine kinase can be induced under cell stress conditions to ensure that the ATP pool is preserved. Pyruvate kinase specific activity displayed a marked increase with NCM and/or ethanol treatment, but this was not statistically significant (**Figure 5A**). However, the specific activity of creatine kinase was elevated by almost 2-fold following NCM treatment in the absence or presence of ethanol (OWH and OEH, respectively) (**Figure 5A**). We next measured the total multidrug resistant (MDR) pump activity which plays a central role in toxin elimination. Total MDR pump activity was significantly increased in NCM treated rats (OWH and OEH) (**Figure 5A**). Treatment with ethanol alone (OEV) had no effect on total ATPase activity. The change in ATP generation and utilization (*e.g.* increased use of ATP to eliminate toxins) prompted us to examine whether other ATPases may be affected by NCM and/or ethanol exposure. We specifically measured the protein levels of ABCA1, an enzyme which hydrolyzes ATP to export cholesterol for the lipidation of ApoA-1 and ApoE and the genesis of HDL. NCM exposure (OWH) induced a substantial decrease (∼2-fold) in ABCA1 protein levels when compared to OWV control rats (**Figure 5B**). Intriguingly the combination of NCM and ethanol decreased ABCA1 levels even further (∼9-fold relative to OWV and OEV). Ethanol alone did not affect ABCA1. We followed up on this observation by immunoblotting for ApoB-100 in obese JCR rats treated with or without NCM and/or ethanol. A trend of decrease, but not to a significant degree, in ApoB-100 levels was observed. We also measured CD36 and L-FABP protein levels. NCM treatment significantly decreased hepatic CD36 and L-FABP levels (**Figure 5C**). The decreases in hepatic fatty acid and cholesterol transporters were accompanied by decreased serum cholesterol and triglyceride levels with or without co-exposure to ethanol (**Figure 5D**).

**Table 4:** Hepatic adenylate and TCA cycle metabolite levels in obese JCR rats dosed with vehicle or NCM and treated with or without ethanol.

	ATP nmol g tissue^−1^	ADP nmol g tissue^−1^	AMP nmol g tissue^−1^	ADP:ATP	AMP:ATP	Citrate nmol g tissue^−1^	2-oxoglut nmol g tissue^−1^
OWV	601.17±67.87	1086.36±122.16	4331.61±414.34	7.33±0.47	1.82±0.13	10.65±1.29	3.02±0.80
OWH	871.90±128.92^a^	734.99±98.01^a^	3140.53±223.26	3.96±0.56^a^	0.85±0.02^a^	8.99±1.23	2.17±0.53
OEV	632.49±83.01	1083.61±148.69	4343.82±756.14	6.84±0.67	1.71±0.07	9.44±1.18	2.89±0.59
OEH	1256.25±154.97^a^	1007.46±97.95	4530.33±460.60	3.66±0.17^a^	0.81±0.001^a^	10.88±1.32	2.21±0.31

*n* = 7–8, means ±SEM, 2-way ANOVA with Tukey's post-hoc test.

a denotes statistical significance at P<0.001 between vehicle (V) control and high dose (H).

## Discussion

NAFLD has become the most prevalent liver disease in North America. In Canada, it is estimated that 25% of people with obesity have non-alcoholic steatohepatitis (NASH), the more advanced form of NAFLD [23]. It has also been reported that approximately 50% of type 2 diabetics and 76% of people with obesity also have fatty liver disease [23]. Rates of liver disease are anticipated to be higher among Northern populations due to alcohol consumption patterns and higher incidence of obesity and type 2 diabetes. It has also been firmly established that prolonged exposure to industrial chemicals and contaminants can result in NAFLD and NASH and that Northern populations can be exposed to high amounts of environmental toxins for prolonged periods [15], [24]. In the present study, we used male obese JCR rats treated with or without NCM in combination with 10% ethanol to determine if *1*) NCM can exacerbate NAFLD in obese rats, *2*) chronic ethanol treatment has an additive effect with NCM exposure, and *3*) NCM and/or ethanol treatment has impact on hepatic energy metabolism and function. This is the first study that attempts to ascertain the collective impact of 22 different contaminants found regularly in Inuit blood on liver physiology and biochemistry and whether or not NCM toxicity can be exacerbated by alcohol exposure and/or obesity. It is important to note though that we utilized a genetically obese rat model to simulate obesity which is not representative of diet or alcohol induced obesity and thus may present a limitation to this study. The obese JCR rats develop obesity due to over eating as a result of defect in leptin signalling. Although humans don't carry the same mutation in leptin receptor as the obese JCR rats, leptin resistance does occur and is known to be associated with obesity in humans. The NCM dose used in this study is necessary to generate, during a four week exposure period, plasma contaminant levels that are comparable to what are detected in the Arctic human populations who are exposed to these contaminants during a life time. The serum levels of individual organic contaminants were within the range of geometric mean and maximum levels found in the Inuit blood [20], suggesting that the doses and duration of exposure used in this study were appropriate. It is interesting that the rats co-exposed to NCM and ethanol had lower serum and hepatic concentrations of organic contaminants than those dosed with NCM alone, implying that ethanol exposure altered the retention/detoxification of NCM, possibly due to altered expression of cytochrome P450 enzymes and transporters leading to altered distribution, metabolism and excretion of these chemicals.

Livers from control obese JCR rats did contain a number of microvesicular lipid droplets with some macrovesicular droplets. Previous reports have established that the absence of leptin receptor induces hyperlipidemia, hypercholesterolemia, hepatic fat accumulation and NAFLD in corpulent JCR rats [25]. Treatment of obese rats with NCM exacerbated NAFLD as evidenced by the accumulation of macrovesicular lipid droplets, and increased lipid content, and total and esterified cholesterol levels in the liver. Exposure to ethanol alone induced a similar effect. Simultaneous exposure to ethanol and NCM did not induce a further increase in the number of macrovesicular lipid droplets. Further, protein levels of ABCA1, which is required for cholesterol efflux from hepatic tissue and the assembly of nascent HDL, was significantly decreased in hepatic tissue from NCM treated JCR rats. These observations indicate that NCM-induced TASH is associated with a drastic decrease in the expression of proteins involved in lipid and cholesterol efflux. Intriguingly, we also observed that NCM treatment diminished the expression of CD36 and L-FABP, two proteins involved in the uptake of fatty acids from circulation. It is entirely possible that the accumulation of hepatic lipids feeds back and inhibits expression of genes and proteins involved in uptake of fatty acids from circulation. In fact, our gene array analysis reveals that NCM treatment decreases gene expression of sterol regulatory element binding protein-1 (SREBP-1) (data to be published separately), a key regulator of fatty acid synthesis and transport. In addition, we also observed that NCM treatment induces mitochondrial dysfunction. Thus, NCM exposure induces drastic changes in overall lipid homeostasis and metabolism in hepatic tissue which most likely contributes to the exacerbation of steatosis.

In the present study we observed that NCM increases total hepatic cholesterol and cholesteryl esters which was matched by a significant increase in mRNA levels encoding proteins involved in cholesterol biosynthesis (unpublished data), but decrease in the protein levels of several enzymes involved in cholesterol biogenesis including diphosphomevalonate decarboxylase, farnesyl pyrophosphate synthase, and CYP51A1. It is known that cholesterol biosynthesis is regulated by a negative feedback control mechanism at the transcriptional level [26]. Our data suggest a disruption of feedback mechanism associated with NCM treatment, which could be involved in posttranslational modification of the protein. In fact, sterols are known to accelerate ubiquitination and degradation of HMGCoA reductase that controls the biosynthesis of cholesterol [27]. Effects of environmental pollutants on cholesterol synthesis have been reported previously. In one study with time profile analysis revealed that the expression of the HMG-CoA reductase gene exhibits biphasic regulation by PCBs [28]. Initial exposure of female Sprague-Dawley rats to PCBs results in an initial increase in HMG-CoA reductase mRNA followed by a decrease down to normal levels [28]. In addition, various reports have shown that PCBs and other POPs can induce hepatic hypocholesterolemia by interfering with cholesterol biosynthesis [29]–[31] whereas other reports have provided results showing that PCBs, insecticides, and toxic metals induce cholesterogenesis [32]–[34]. Another possibility is that in the NCM-treated rat liver translation of cholesterol biosynthesis mRNA is being impeded by expression of microRNA. Indeed, hepatic cholesterol and fatty acid levels play an intricate role in modulating lipid and cholesterol biosynthesis and degradation via the expression of various microRNAs. For example, miR-122 plays a critical role in the inhibiting expression of mRNA that encodes proteins involved in fatty acid oxidation and cholesterol degradation [35]. In addition, NASH is associated with drastic changes in the levels of various hepatic microRNAs including miR-122 [36]. miR-33 has also been recently identified as a key modulator of cholesterol homeostasis in hepatic tissue [37].

Isoprene units from the cholesterol biosynthesis pathway are required for the biosynthesis of Q [22]. Coenzyme Q_10_ transfers electrons from Complexes I and II and electron-transferring-flavoprotein dehydrogenase- ubiquinone oxidoreductase to Complex III in the respiratory chain. In the present study, we found that NCM substantially decreased geranylgeranyl diphosphate synthase levels, which commits isoprene units to Q biosynthesis. This was matched by a ∼3-fold drop in hepatic Q levels. We also examined the activity of respiratory complexes and the expression of individual subunits for the different respiratory complexes. Most notably, we recorded a substantial decrease in the expression of ATP5A subunit for Complex V, which is required to drive aerobic ATP synthesis. Simultaneous exposure to ethanol and NCM had an additive effect on ATP5A expression. Intriguingly, although the Complex I subunit NDUFB8 was virtually absent in the ethanol treated rats (both in the absence or presence of NCM), the specific activity was unaffected by NCM treatment. It is important to note that NDUFB8 is an accessory protein and is not required for the activity of Complex I. Although very little information exists on the true function of NDUFB8, it has been suggested that NDUFB8 is required for respirasome assembly [38]. Respirasomes are essentially supercomplexes composed of Complexes I, III, IV, and V. Supercomplex assembly is required for efficient electron transfer to molecular oxygen which limits ROS formation by the electron transport chain and enhances ATP formation by Complex V [39]. Deficiencies in supercomplex assembly are associated with diminished respiration and ATP formation by mitochondria [40]. However, loss of supercomplex assembly may not necessarily affect the activity of the individual Complexes. Thus, loss of NDUFB8 would ultimately compromise ATP production by mitochondria. We also noted that Complex IV activity was substantially decreased in liver extracts from obese JCR rats treated with both NCM and ethanol. This decrease was matched by a decrease in mitochondrially encoded cytochrome c oxidase I (MTCO1), a Complex IV subunit [41]. Notably, deficiencies in MTCO1 expression are often associated with mitochondrial DNA damage and development of mitochondrial diseases [42]. PCBs and other contaminants are known to accumulate in mitochondria and can subsequently induce electrophilic damage to mitochondrial DNA, leading to development of mitochondriopathies [19]. Thus, our results demonstrate that exposure to NCM induces mitochondrial dysfunction and depletes Q which would ultimately compromise lipid homeostasis.

Mitochondria are central to maintenance of hepatic lipid homeostasis. Perturbations in mitochondrial respiration can also compromise energy production [43]. However, in spite of the mitochondrial dysfunction, NCM increased hepatic ATP levels. This was due, in part, to an increase in creatine kinase levels. Contaminants like chlordane have been shown to increase creatine kinase in muscle and liver [44], We observed no changes in pyruvate kinase activity however; our gene array analysis revealed alterations in the expression of genes involved in glycolysis, gluconeogenesis, and citrate cycle (data to be published separately), which may help to satisfy cellular ATP demands in response to mitochondrial dysfunction. The increase in hepatic ATP content could also be due to its diminished utilization. Indeed, we observed that ATP consuming transporters like ABCA1 displayed a substantial decrease in expression. This was matched by our gene array results (unpublished data) that showed that the expression of various plasma membrane transporters that couple lipid and/or cholesterol efflux to ATP hydrolysis were also down regulated. It is entirely possible that ATP consuming proteins may also be diminished in an attempt to preserve hepatic ATP pools for detoxification reactions. Indeed, we observed that the ATPase activity of various MDR pumps was increased in liver extracts from JCR rats. MDR pumps play a central role in xenobiotic elimination, a process that requires hydrolysis of ATP. In addition, we also observed that NCM increased CYP2E1, a key enzyme involved in catalysis of phase I detoxification reactions. Further, detoxification reactions catalyzed by phase I and phase II enzymes in hepatic tissue require a steady input of ATP. Thus, the observed shift in hepatic ATP homeostasis in NCM treated JCR rats is required to preserve ATP pools for xenobiotic detoxification and elimination.

Obesity, prolonged exposure to environmental contaminants, and alcohol abuse lead to development of NAFLD which is characterized by mitochondrial dysfunction, diminished β oxidation, and an accumulation of hepatic lipids. Chronic NAFLD can lead to NASH, which is characterized by liver failure. Few studies have actually investigated whether contaminant exposure can have an additive effect with existing obesity on NAFLD and whether or not chronic alcohol abuse can exacerbate these effects further. This is especially relevant for Northern populations since they suffer from increasing obesity rates, are exposed to elevated concentrations of contaminants, and have higher population rates of alcohol abuse. Here, we show that NCM exacerbates hepatic steatosis in genetically obese male JCR rats. These effects were associated with substantial shifts in lipid homeostasis and transport which resulted in an accumulation of cholesterol and cholesteryl esters and alterations in fatty acid profile. In addition, we also observed that NCM induced a substantial shift in ATP metabolism, mitochondrial dysfunction, and an upregulation of proteins involved xenobiotic detoxification. Collectively, our findings indicate that environmental contaminants found in Northern Canada can worsen an existing hepatic steatosis associated with obesity. Future studies utilizing rats fed high fat/high sugar diets will be performed to simulate the impact of diet-induced obesity on NCM toxicity in hepatic tissue.

## Materials and Methods

### Animals

All animal work was conducted according to the guide line of Canadian Council on Animal Care (CCAC). The protocol (HCO-ACC-2010-020) was approved by the Animal Care Committee of Health Canada. All animals were housed separately in plastic cages with wood chip bedding in the animal care facility of Health Canada. The rooms were ventilated and maintained at constant temperature of 22±2°C and humidity of 50% with a 12/12 h light/dark cycle. Food and water were given ad libitum. Serological testing was conducted and results were all negative. Animals were euthanized using isoflurane at the end of the study.

Obese JCR (LA)-Leprcp (cp/cp) male rats at age of 8 weeks were obtained from Metabolic and Cardiovascular Diseases Laboratory (University of Alberta, Edmonton, Alberta, Canada) and used as model systems. The cp/cp rats are homozygous for the autosomal recessive cp gene, resulting from the Tyr763Stop mutation for the leptin receptor (ObR). Without ObR, these rats become obese, hyperlipidemic, and hyperinsulinemic [45], [46]. Thus, the cp/cp male rat is an ideal model for determining if NCM dosing exacerbates the co-morbidities associated with obesity.

Animals were fed an AIN93G purified casein-based diet for 2 weeks, and then acclimatized to cookies for another 8 weeks. Note that it was necessary to acclimatize the animals to a cookie diet prior to dosing since the NCM was dried on cookies prior to dosing for ingestion. From the third week of cookie treatment, the animals were divided into two groups and dosed with 1% (v/v) ethanol and additional 1% every other day until 10% ethanol in drinking water for two weeks followed by dosing with 10% (v/v) ethanol from week 5 until the end. Water served as the control for the ethanol dosing. JCR rats were dosed for a total of 6 weeks with ethanol. From the third week of ethanol treatment, the animals in each ethanol dose group were divided again into two groups and dosed daily with vehicle solvent (corn oil) or 16 mg NCM/kg BW for a total of 4 weeks until the end of the study. Animal weights, and food and water consumption were measured daily. At the end of the study, animals were euthanized and serum collected for analysis. Livers were weighed, separated, and placed in either 10% buffered formalin for histology or snap frozen in liquid nitrogen and stored at −80°C for further analysis. All animal studies were conducted in accordance with the guideline of Canadian Council on Animal Care. AIN93G purified diet consisted of 17.7% protein (casein), 7.2% fat (soybean oil), 5.0% fiber (cellulose), and 60.1% carbohydrates (corn starch) (Harlan Laboratory, Madison, WI, USA). AIN93G purified diet consisted of 18.3% protein, 7.1% fat, 5.0% fiber, and 63.2% carbohydrates (Testdiets). Linoleic acid (LA) and α-Linolenic acid (ALA) accounted for 3.58 and 0.55%, respectively, of the fatty acids. No arachidonic acid (AA) was present in the diets. Saturated fatty acids (SFA) and monounsaturated fatty acids (MUFA) made up 1.05 and 1.54% of the fatty acids respectively.

### Serum metabolites

Serum cholesterol and triglycerides were measured using the Hitachi Model 917 Multichannel Analyzer (Biomedical Lab Center). Assays were conducted as described by the manufacturer.

### Pathology/Histology

A slice of liver tissue was fixed in 10% buffered formalin, and embedded in paraffin wax. Sections of 5 µm thickness were cut on microtome, deparaffinised, and stained with hematoxylin and eosin. Pathological changes were examined under light microscope with a 20× objective. A subjective numeric grading from 0 to 5 was used to record lesions. Macrovesicular lipid droplet number and diameter were determined using Axiovision Rel. 4.6 software (Zeiss).

### Hepatic and serum content of contaminants

Pulverised liver tissue (0.2∼0.5 g) and serum samples were weighed and extracted with 3 mL of acetone and 2+2 mL of aceone:hexane (2∶1). The sample extract was combined and concentrated with nitrogen stream to a volume of 2.5 mL, and re-extracted three times with 3, 1.5, and 1.5 mL of dichloromethane (DCM). The sample was laid in fume hood overnight to let the solvent to evaporate to dry. The sample was weighed and the lipid content was measured gravimetrically. The sample was re-dissolved in one mL of 30% DCM in hexane and 30 µL methanol. Derivatization of pentachlorophenol and tetrabromophenol was conducted by adding 30 µL of 2 M trimethyldiazomethane solution in hexane. The sample was dried and redissolved in 1 mL 30% DCM in hexane and cleaned with a column packed with 3.0 g 2% deactivated Florisil. The analytes were eluted with 30 mL of 30% DCM in hexane. The sample was concentrated with a rotavapor and nitrogen stream to a final volume of 0.5 mL prior to analysis with gas chromatography (GC). The GC analysis was conducted with an Agilent 7890A GC equipped with electron capture detector.

### Hepatic total, free, and esterified cholesterol

Liver cholesterol levels were determined using Cholesterol/Cholesteryl Ester Quantification Kit from Abcam Inc. (Toronto, Ontario, Canada). Briefly, pulverized liver tissue of 30–50 mg was extracted in 600–1000 µl chloroform:isopropanol:NP-40 (7∶11∶0.1) using Coveris S2 sonicator (Covaris, Inc, Woburn, Massachusetts, USA). Tissue extract of 400 ul was transferred to a 1.5 ml tube and spun at 15000 g for 5 min. Organic phase was transferred to another 1.5 ml tube and dried under nitrogen stream. Dried extract was dissolved in 400 µl assay buffer provided with the kit. This sample solution was diluted 20 times and assayed as described by the manufacture.

### Hepatic CD36 and L-FABP

Pulverized frozen liver samples were homogenized in cold PBS buffer using Coveris S2 sonicator. Protein extracts were centrifuged at 5000 g and 4°C for 5 min. Supernatants were analyzed for protein concentrations using DC Protein Assay (BioRad) and for levels of fatty acid translocase (CD36) and liver fatty acid binding protein (L-FABP) using ELISA kits from MyBioSource after 4000–6000× dilution in sample dilution buffer.

### Hepatic fatty acid levels

Pulverized frozen liver samples (1 g) were placed in a glass tube containing 10 mL chloroform:methanol (2∶1) and homogenized using a Polytron PT 1300 D at 5000 rpm for 1 min. Liver homogenates were then vacuum-filtered through Whatman paper No1. The eluent was then salted out by adding 6 mL of 0.58% (w/v) NaCl solution. Samples were then mixed by swirling and centrifuged at 2000 rpm for 5 min at room temperature. The upper phase was aspirated and the lower was treated with Na_2_SO_4_ and then incubated at room temperature for 10 min. Samples were then transferred into pre-weighted tubes and solvents were then evaporated under nitrogen gas at 40°C for 1–3 h. Once dry, tubes were weighed for percentage of recovery, and FFA was resuspended in 1 mL of Hexane. Derivitization of FFA to methyl ester (FAME) was done using Boron trifluoride methanol solution. FFA (15 mg) was incubated for 1 h at 105°C in 1∶1 (v/v) toluene and boron trifluoride in 7% methanol (w/w) (Sigma-Aldrich B1252). FAMEs were isolated with several hexane and deionized water cleaning and dried with granular sodium sulfate salt, resuspended in hexane, and analysed using GC-FID instrument at 21× dilution.

FAMEs (2 ul split) were analysed by GC with a flame ionization decay detector and auto-sampler (Agilent Technologies Canada Inc., Mississauga, ON, Canada) using a biscyanopropyl column (Supelco, SP-2560, L × I.D. 100 m×0.25 mm, df 0.20 µm, Sigma-Aldrich Canada Co., Oakville, Ontario, Canada). The fatty acids were identified by comparing their retention times with those of a standard mixture of FAMEs (Supelco 37 FAME catalogue number 47 885-U; Supelco Inc.). The fatty acids were expressed as % of total fatty acids.

### Cholesterol biosynthesis enzyme levels

Analysis was conducted on pulverized liver samples that were snap frozen and stored at −80°C. Snap frozen liver samples (0.1–0.2 mg) were immersed in liquid nitrogen for 15 seconds and then pulverized using the Covaris CP-02 cryoprep system (Covaris). Samples were immersed in liquid nitrogen for an additional 10 seconds and the sample was then placed in a Covaris sample collection tube. The pulverized samples were then resuspended in 1 mL of 1× RIPA buffer containing Halt Protease Inhibitor (100 µl Halt in 50 mL of RIPA, Pierce), vortexed, and incubated on ice for 30 min. Samples were then centrifuged at 10,000xg for 10 min at 4°C and the resulting supernatant was aliquoted in 100 µl and stored at −20 °C. The enzyme protein levels of 3-hydroxy-3-methyl glutarate (HMG-CoA), 3-hydroxy-3-methyl glutyryl CoA synthase (HMG-CoA synthase), diphosphomevalonate decarboxylase, farnesyl pyrophosphate synthase, geranylgeranyl pyrophosphate synthase, and lanosterol 14α-demethylase were assayed using ELISA kits purchased from CUSABIO. All assays were conducted according to manufacturer's instructions except all incubations were performed at room temperature rather than 37°C. Prior to each assay, protein concentrations for each sample were adjusted to 1–4 mg/ml. Color changes were measured using a POLARstar Optima 96-well plate reader (BMG Labtech) and all analysis was conducted using Optima software (BMG Labtech). Protein concentrations were determined by DC assay (BioRad) according to manufacturer's instructions using defatted BSA as the protein standard.

### HPLC analyses

Liver samples (0.1–0.3 mg) were weighed and pulverized as described above. Adenylate measurements were performed as described in [47]. For measurement of absolute levels of adenylates (ATP, ADP, and AMP) and organic acids (citrate and 2-oxoglutarate), pulverized livers were treated with 1 mL of 0.5% (v/v) perchloric acid solution (prepared in mobile phase), vortexed vigorously for 30 seconds, and then incubated on ice for 10 min. Samples were then centrifuged at 10,000xg for 10 min at 4°C and the resulting supernatant was filtered through 0.2 micron filters directly into HPLC sampling vials. Samples were injected into a Finnigan Surveyor HPLC system (Thermo Fisher) equipped with a C_18_ Synergi Reverse Phase column (Phenomenex, 4 µ Hydro-RP, 250×4.6 mm) and Security Guard Column (Phenomenex) operating at a flow rate of 0.7 ml/min. All injections were performed at ambient room temperature. The mobile phase was 20 mM KH_2_PO_4_ (pH 2.9). Adenylates and organic acids were measured simultaneously at 254 and 210 nm respectively. Absolute amounts of adenylates and organic acids were estimated using ChromQuest 4.1 software and by injecting various amounts of standard solutions. Integration values for peaks corresponding to the individual adenylates and organic acids were then used to calculate nmol amounts of metabolites. All values were normalized to grams of tissue.

Measurement of Coenzyme Q_10_ (CoQ_10_) was performed as described in [48]. Pulverized liver samples were resuspended in 1 mL of ice cold 1-propanol, vortexed vigorously for 30 seconds, and then incubated on ice for 5 min. Samples were then vortexed vigorously for 30 seconds and centrifuged at 10, 000 xg for 10 min at 4°C. The supernatants were then collected and filtered through 0.2 micron filters directly into HPLC sampling vials. Samples were injected into Finnigan Surveyor HPLC system (Thermo Fisher) equipped with a Luna 5u C_18_ column (150×4.6 mm, Phenomenex) operating at a flow rate of 1 ml/min and 25°C. The mobile phase was 65% HPLC grade ethanol and 35% HPLC grade methanol. Adenylates and organic acids were measured simultaneously at 254 and 210 nm respectively. Absolute amounts of CoQ_10_ were estimated using ChromQuest 4.1 software and by injecting various amounts of standard solutions. Integration values for peaks corresponding to CoQ_10_ were then used to calculate nmol amounts of metabolites. All values were normalized to grams of tissue.

### Creatine kinase activity

Creatine kinase activity was assayed by HPLC. Pulverized liver samples were treated with liver homogenization (LH) buffer (220 mM mannitol, 70 mM sucrose, 20 mM HEPES, 1 mM EGTA, pH 7.4–1% (w/v) defatted BSA and 10 mM DTT added fresh on the day of experiment) containing 1% (v/v) β-dodecyl-D-maltoside, vortexed for 5 seconds, and then incubated on ice for 30 min. Samples were then centrifuged as described above and the resulting supernatant was assayed for creatine kinase activity. Briefly, protein samples were 0.1 mg/mL in LH containing creatine (0.1 mM) and ADP (0.1 mM) in a final volume of 1 ml. Reactions were then allowed to proceed for 5 min and then stopped by adding 50 µL of ice cold 0.5% (v/v) perchloric acid solution followed by a 10 min incubation on ice. Samples were clarified by centrifugation and filtration and the amount of ADP consumed in the reaction was quantified as described above. The specific activity of creatine kinase was calculated as the amount of ADP consumed over the 5 min period divided by the amount of protein used for the reaction. Samples devoid of protein were used as a control and activities were calculated based on the amount of ADP in the reaction at time 0.

### Respiratory complex and pyruvate kinase activities

Enzyme activities were measured in liver samples treated with LH and β-dodecyl-D-maltoside. Complex I activity was measured as described in [49]. Proteins were diluted to 0.1 mg/mL LH containing KCN (2 mM) and antimycin A (0.3 µM). Reactions were initiated by addition of NADH (0.1 mM). Complex IV was monitored as described in [50]. Briefly, protein was diluted to 0.1 mg/mL LH and then reactions were initiated by addition of reduced cytochrome C (30 µM). Pyruvate kinase was monitored as described in [51]. Protein samples were diluted to 0.1 mg/mL LH containing ADP (0.6 mM), MgCl_2_ (5 mM), and exogenous lactate dehydrogenase (8 units). Reactions were initiated by simultaneous addition of phosphoenolpyruvate (PEP, 0.5 mM) and NADH (0.2 mM). All reactions were monitored for a total of 5 min. Activities were normalized to measurements performed in the absence of NADH or cytochrome C. Activities were calculated using molar extinction coefficients ε_NADH_ = 6,220 M^−1^ cm^−1^ and ε_Cyt C_ = 18.5 mM^−1^ cm^−1^. Rates of NADH and cytochrome C consumption were calculated 30 seconds into the reaction (which represents the fastest consumption of either substrate).

### Total MDR pump ATPase assay

The activities of MRP 1, MRP 2, MRP 3, BCRP, and MDR 1/P-gp were assayed using the BD Gentest ATPase Assay kit (BD Biosciences, Woburn, MA) as described by the manufacturer.

### Immunoblots

Liver samples were diluted to 2 mg/mL in Laemmli buffer containing 2% (v/v) β-mercaptoethanol and then heated at 100°C for 5 min. Protein samples were electrophoresed in 10% isocratic SDS-gels followed by electroblotting to nitrocellulose membranes. Nonspecific binding sites were blocked by incubating membranes at room temperature under constant agitation with TBS-T (Tris-buffered saline (pH 8.0) +0.1% (v/v) Tween-20) solution containing 3% (w/v) nonfat skim milk and 2% (v/v) fetal bovine serum. After two washes with TBS-T, membranes were incubated overnight at 4°C in primary antibodies directed against respiratory complex subunits (MitoProfile OXPHOS antibody, Abcam, 1/1,000, 20 µg sample loaded), Cytochrome P450 2E1 (CYP2E1, Santa Cruz, 1/1,000, 10 µg sample loaded), ATP binding cassette transporter 1 (ABCA1, 1/1,000, 30 µg sample loaded), or apolipoprotein B-100 (ApoB-100, 1/1,000, 30 µg sample loaded). Glyceraldehyde-3-phosphate dehydrogenase (GAPDH) served as the internal loading control (Sigma-Aldrich, 1/10,000 dilution). The Mitoprofile antibody detects specific subunits for the different respiratory complexes simultaneously; NDUFB8 (Complex I), SDHB (Complex II), UQCRC2 (Complex III), MTCO1 (Complex IV), and ATP5A (Complex V). Also, the MitoProfile antibody was diluted in TBS-T containing 5% (w/v) defatted bovine serum albumin and 0.005% NaN_3_ while the other antibodies were diluted in blocking solution. Antibody exposures and dilutions were optimized prior to experimentation. Membranes were then probed with the requisite secondary antibody HRP conjugate (Dako). Membranes were visualized using SuperSignal West Pico Chemiluminescent Substrate (Thermo Scientific).

### Statistical analysis

All statistical analysis was carried out using SigmaPlot Software (version 11.0). Statistical comparisons were performed using 2-way ANOVAs with Tukey's post-hoc test. Nonparametric data were transformed or ranked prior to statistical analysis. For all statistical tests p values were set to 0.05.
